# NAMPT shows tissue-dependent immune-metabolic associations in osteoarthritic synovium and aging-related skeletal muscle

**DOI:** 10.3389/fmed.2026.1853301

**Published:** 2026-07-28

**Authors:** Qianyu He, Weikai Qin, Ximing Yu, Lin Jing, Qi Yan

**Affiliations:** Wangjing Hospital, China Academy of Chinese Medical Sciences, Beijing, China

**Keywords:** external support, immunometabolism, knee osteoarthritis, NAD salvage, sarcopenia, skeletal muscle aging, synovitis

## Abstract

**Introduction:**

Nicotinamide phosphoribosyltransferase (NAMPT) is a key immune-metabolic regulator linking nicotinamide adenine dinucleotide (NAD) salvage, inflammatory signaling, and tissue stress responses, but whether it occupies comparable transcriptomic contexts in osteoarthritic synovium and skeletal muscle remains unclear.

**Methods:**

We performed a retrospective multi-cohort transcriptomic analysis using public GEO datasets with separate discovery and external supporting analysis stages. Discovery analyses included knee osteoarthritis synovium cohorts GSE55235 and GSE55457 and aging-related skeletal muscle proxy cohorts GSE38718 and GSE25941. Muscle-side findings were interpreted with explicit separation between aging-related skeletal muscle proxy cohorts and the clinically phenotyped sarcopenia external-support cohort. NAMPT abundance was evaluated by differential expression analysis and effect-size synthesis, immune and metabolic pathway activity was quantified by single-sample gene set enrichment analysis, and disease-adjusted partial correlations were used to define discovery-stage association structure. External supporting analyses were then performed in GSE12021, GSE254682, and the clinically phenotyped sarcopenia cohort GSE111016 to assess whether the direction of NAMPT abundance differences was reproduced in independent disease-relevant cohorts.

**Results:**

Across the discovery stage, NAMPT showed a downward abundance signal in both tissue contexts, although meta-analytic pooling in knee osteoarthritis synovium was limited by between-study heterogeneity. External datasets provided partial support for lower NAMPT expression in disease-relevant synovium in GSE12021 and in clinically phenotyped sarcopenia muscle in GSE111016, whereas GSE254682 showed the same direction without statistical significance. Discovery-stage disease-adjusted coupling analyses indicated that osteoarthritic synovium retained stronger NAMPT associations with inflammatory and glycolytic programs, whereas aging-related skeletal muscle proxy cohorts showed weaker immune coupling and a pattern more consistent with metabolic deficit. Cohort-stratified stability analyses suggested that these associations were directionally consistent in osteoarthritic synovium but weaker and more heterogeneous in the muscle proxy cohorts. A reduced external association analysis showed partial module-level consistency with this contrast, including preserved inflammatory linkage in GSE254682 and a more oxidative-phosphorylation-oriented pattern in GSE111016. Exploratory cohort-level separation analyses and therapeutic inference were retained as secondary analyses and were not interpreted as evidence of clinical utility or efficacy.

**Discussion:**

These findings suggest reduced NAMPT abundance with partial external support together with observational discovery-stage evidence of tissue-dependent immune-metabolic association patterns, and they favor context-specific interpretation and targeted experimental follow-up rather than uniform extrapolation across tissues.

## 1 Introduction

Knee osteoarthritis (KOA) and skeletal muscle decline in older adults are closely linked clinically, but they arise in biologically distinct tissue environments. KOA is now recognized as a whole-joint disorder in which synovitis, stromal activation, and immune cell infiltration contribute directly to pain and structural progression (1–7). Skeletal muscle aging, by contrast, is characterized more by loss of metabolic flexibility, mitochondrial resilience, and proteostatic control, with inflammatory signals often superimposed on a background of energetic stress rather than a uniformly inflamed tissue niche (8–16). This distinction matters because superficially similar transcriptomic signals across tissues may reflect very different biological states.

*NAMPT* is particularly informative at this interface. As the rate-limiting enzyme in the mammalian NAD salvage pathway, intracellular *NAMPT* supports bioenergetic homeostasis, sirtuin activity, and stress adaptation, whereas extracellular *NAMPT* can act as an inflammatory mediator in several pathological settings (17–27). This dual role is especially relevant in musculoskeletal disease. OA-related studies often emphasize visfatin/e*NAMPT* in inflammatory fluids, whereas studies of muscle biology and aging have focused on intracellular *NAMPT*-driven NAD salvage in maintaining mitochondrial function and contractile capacity (24, 25, 28–30). An interpretation based only on overall abundance therefore risks collapsing biologically distinct compartments into a single narrative.

Recent studies have further refined NAMPT/NAD biology in directions relevant to the present analysis. Updated reviews emphasize the central role of NAMPT in NAD+ salvage, cellular stress adaptation, aging, and metabolic disease, whereas eNAMPT-focused work highlights that extracellular NAMPT has inflammatory and biomarker functions that are not equivalent to intracellular transcript abundance (26, 27). In parallel, recent studies of skeletal muscle aging have renewed interest in NAMPT-driven NAD+ salvage and NAD restoration as regulators of mitochondrial function, metabolic flexibility, and muscle maintenance (30, 31).

A substantial literature has described changes in *NAMPT* abundance in osteoarthritic tissues, systemic inflammatory states, and aging muscle. What remains unresolved is whether *NAMPT* occupies the same immune-metabolic association structure across tissues. This issue is further complicated on the muscle side by phenotype definition: age-only cohorts, aging-related transcriptomic proxy cohorts, and clinically phenotyped sarcopenia cohorts should not be treated as interchangeable. A signal that appears reproducible in clinically defined sarcopenia may be less stable in datasets that mainly capture chronological aging or mixed exercise-related biology.

We therefore framed this study around tissue context rather than single-gene classification. Our aims were to characterize the discovery-stage *NAMPT* abundance signal, determine whether *NAMPT* shows context-dependent immune-metabolic associations in KOA synovium and skeletal muscle transcriptomic settings, add an external supporting analysis layer using disease-relevant synovium and a clinically phenotyped sarcopenia cohort, and generate cautious, hypothesis-generating interpretations for future experimental investigation.

## 2 Materials and methods

### 2.1 Study design and cohort definition

**Figure 1:**
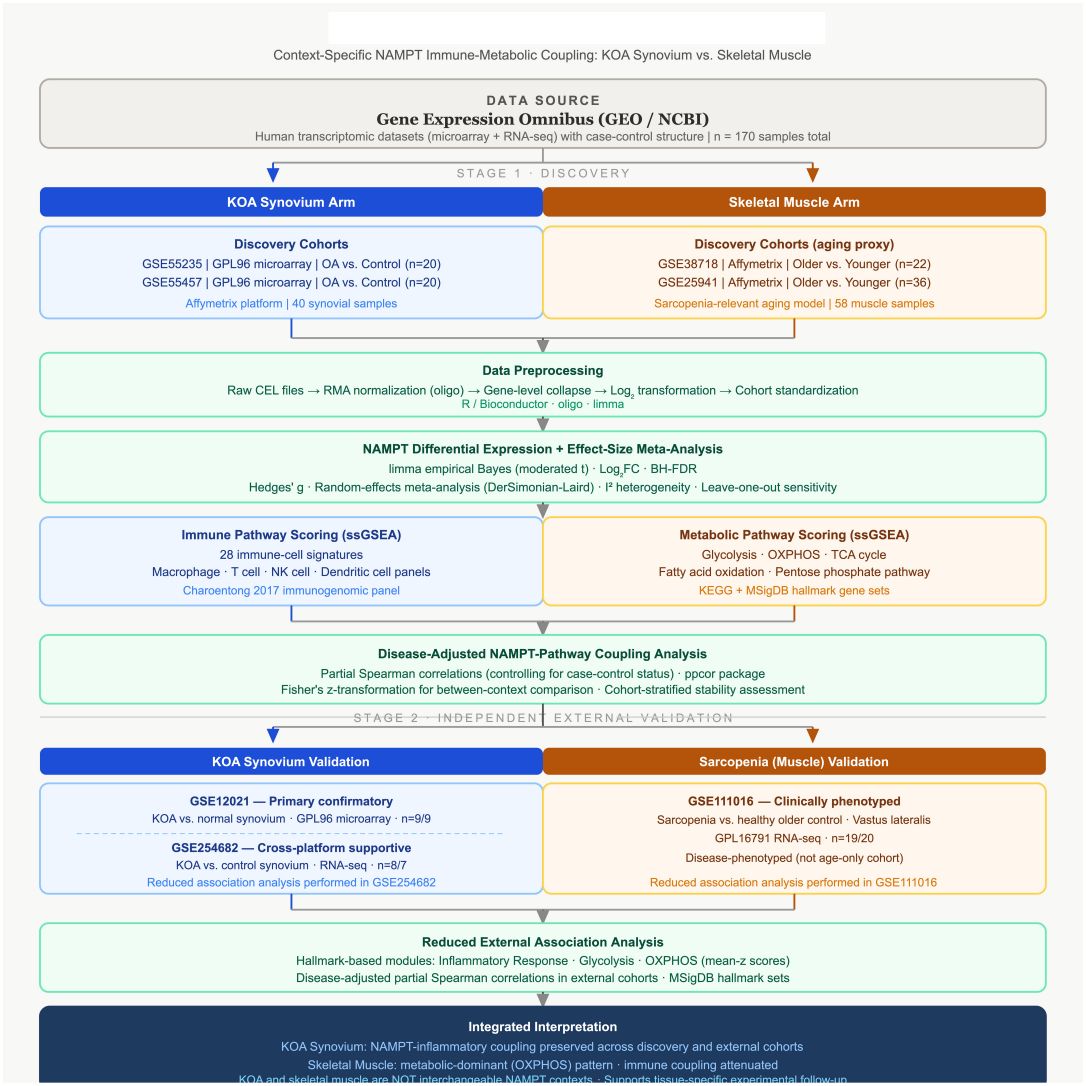
Study design and analytical workflow. Public GEO transcriptomic cohorts for KOA synovium and skeletal muscle transcriptomic contexts were analyzed in parallel. The workflow summarizes discovery-stage differential expression profiling, external cohort support analysis, immune and metabolic pathway scoring, disease-adjusted association analysis, and exploratory therapeutic inference

This study used a retrospective public-data design centered on *NAMPT* across two tissue contexts. The study design and analytical workflow are summarized in Figure 1. The analytical workflow was divided into a discovery stage and an external supporting analysis stage. Discovery analyses were intended to characterize abundance signals, immune and metabolic landscape differences, and disease-adjusted association structure. The external supporting analysis was intended to assess directionality and group-level consistency in independent public cohorts rather than to establish clinical utility. All hypothesis-testing analyses were performed within cohort, whereas cross-cohort displays use standardized summaries rather than merged, batch-corrected inference. The synovial arm focused on KOA-relevant tissue. The muscle-side analysis was explicitly divided into aging-related skeletal muscle proxy cohorts in the discovery stage and a clinically phenotyped sarcopenia cohort in the external support stage. This distinction was maintained throughout the manuscript to avoid treating chronological aging-related transcriptomic changes as equivalent to clinically defined sarcopenia pathology.

### 2.2 Discovery cohorts

Discovery cohorts were retrieved from GEO using predefined inclusion criteria: human samples, disease-relevant tissue, case-control structure, and accessible expression matrices with adequate sample size (32). KOA discovery cohorts comprised GSE55235 and GSE55457, both synovium-based microarray datasets generated on GPL96 and together including 20 OA and 20 control samples. Discovery skeletal muscle cohorts comprised GSE38718 and GSE25941, which were retained as aging-related skeletal muscle proxy cohorts. GSE38718 included 8 older and 14 younger adults, whereas GSE25941 included 21 older and 15 younger women. These cohorts were interpreted conservatively as aging-related skeletal muscle transcriptomic proxy cohorts rather than clinically confirmed sarcopenia cohorts, in line with the original dataset descriptions and prior cohort-level transcriptomic reports (33–35).

### 2.3 External supporting cohort analysis

External supporting cohorts were selected to provide an additional disease-relevant comparison layer aligned with the revised framing of the manuscript. GSE12021 was used as an external-support KOA synovium microarray cohort, GSE254682 as a cross-platform supportive KOA synovium RNA-seq cohort, and GSE111016 as an independent clinically phenotyped sarcopenia cohort. GSE111016 was derived from the Singapore Sarcopenia Study reported by Migliavacca et al. and was treated as a clinically phenotyped sarcopenia cohort. In the original study, sarcopenia was defined according to the Asian Working Group for Sarcopenia criteria as appendicular lean mass index normalized for height ≤ 7.00 kg/m^2^ together with either low physical performance based on gait speed ≤ 0.8 m/s or low muscle strength based on hand grip < 26 kg. Because these cohorts differed in platform and data structure, they were not used for direct cross-platform quantitative comparison.

Instead, the external support layer was explicitly defined as an assessment of direction and group-difference replication. *NAMPT* expression was extracted in cohort-specific units from the deposited processed matrices or the series-level normalized tables available in GEO. The purpose of this external support analysis was to determine whether the abundance signal remained supported in disease-relevant synovium and clinically defined sarcopenia, not to claim formal clinical utility. To assess the generalizability of the NAMPT-related findings, we examined external transcriptomic datasets. These cohorts provided external support for the directional downregulation of NAMPT at the group level, although differences in platform, sample characteristics, and available features precluded a full replication of the discovery-stage association architecture.

### 2.4 Data preprocessing

For discovery microarray cohorts, raw CEL files were processed in R using oligo and Robust Multi-array Average background correction, quantile normalization, and probe summarization (36–38). Probe sets were collapsed to gene-level expression using platform-specific annotation and a maximum-average strategy when multiple probes mapped to the same symbol. Expression values were log_2_ transformed and standardized within cohort for between-dataset visual comparison, whereas all differential analyses were performed within individual cohorts. *NAMPT*-focused summary statistics and full differential expression outputs are provided in Supplementary Table 1.

For the external supporting cohorts, submitter-provided normalized values were retained in their original cohort-specific units. Because the external support objective was to assess within-cohort direction and group-level differences, no cross-platform rescaling or merged analysis was performed.

### 2.5 Differential expression and effect-size synthesis

Discovery-stage differential expression analysis was performed with limma using disease status or older-versus-younger group as the primary design factor (39). Moderated statistics, log2 fold changes, and adjusted *P* values were calculated using empirical Bayes moderation and Benjamini–Hochberg false discovery rate control. *NAMPT*-centered effect-size synthesis was performed using Hedges' g and random-effects meta-analysis to assess consistency across datasets within each tissue context, with heterogeneity assessed by Cochran's Q and I2 statistics (40–42). Complete differential expression outputs and *NAMPT*-centered meta-analytic summaries are provided in Supplementary Table 1.

In the KOA arm, substantial between-study heterogeneity was treated as a reason to avoid overinterpreting pooled estimates. In the aging-related skeletal muscle proxy arm, pooled effect estimates were reported to summarize consistency across the two proxy cohorts.

### 2.6 Immune and metabolic pathway scoring

Single-sample gene set enrichment analysis, implemented with GSVA-derived ssGSEA scoring, was used to quantify immune-cell signatures and metabolic pathway activity in each discovery cohort (43). Immune scoring used a curated panel of 28 immune-cell signatures derived from prior immunogenomic studies (44), whereas metabolic scoring focused on glycolysis, oxidative phosphorylation, tricarboxylic acid cycle activity, fatty acid metabolism, pentose phosphate pathway activity, and related pathway sets derived from KEGG and MSigDB (45, 46). Disease-versus-control feature comparisons are summarized in Supplementary Table 3, and downstream functional enrichment context is provided in Supplementary Table 2.

Scores were compared between case and control groups within each cohort. The purpose was to characterize the transcriptomic context surrounding *NAMPT* abundance changes rather than to infer absolute cell fractions or metabolite flux.

### 2.7 Disease-adjusted coupling analysis

To distinguish intrinsic association structure from simple group separation, *NAMPT*-pathway relationships were analyzed using disease-adjusted partial Spearman correlations implemented with the ppcor framework (47). Only disease/group status was prespecified as the adjustment variable so that group-separation effects could be reduced without conditioning on downstream post-outcome covariates. Differences in correlation strength between tissue contexts were examined by Fisher's z transformation, and heatmaps were visualized with ComplexHeatmap (48). Full correlation outputs are provided in Supplementary Table 4, and the contrast between unadjusted and disease-adjusted methods is shown in Supplementary Figure 3.

Throughout the manuscript, these results are interpreted as disease-adjusted association structure or context-dependent coupling, not as direct evidence of causality or experimentally established mechanism.

For discovery-stage stability assessment, cohort-stratified disease-adjusted partial correlations were summarized for prespecified immune and metabolic features. Approximate 95% confidence intervals for partial correlation coefficients were derived by Fisher's z transformation assuming one adjustment variable (disease status), and these values were used to display directional consistency and uncertainty across the four discovery cohorts.

To examine only a limited set of core directions outside the discovery datasets, a reduced external association analysis was performed in GSE254682 and GSE111016 using hallmark-based inflammatory response, glycolysis, and oxidative phosphorylation modules summarized as within-cohort mean-z scores. Disease-adjusted partial Spearman correlations were then estimated between *NAMPT* and each module while controlling for case-control status. Because the external cohorts did not support full harmonization of the discovery-stage feature framework, this reduced external association analysis was interpreted as a limited assessment of selected module-level directions rather than as validation of the full discovery-stage immune-metabolic association architecture.

### 2.8 Exploratory cohort-level separation analysis

Receiver operating characteristic analysis was retained as a secondary descriptive analysis to summarize within-cohort separation of *NAMPT* between groups. AUC values and bootstrap distributions were generated using pROC, with DeLong-based comparison used where appropriate (49, 50). These analyses are presented as exploratory cohort-level separation analyses and were not intended as evidence of clinical utility; the corresponding outputs are shown in Supplementary Figures 1 and 2.

### 2.9 Exploratory therapeutic inference

**Figure 2:**
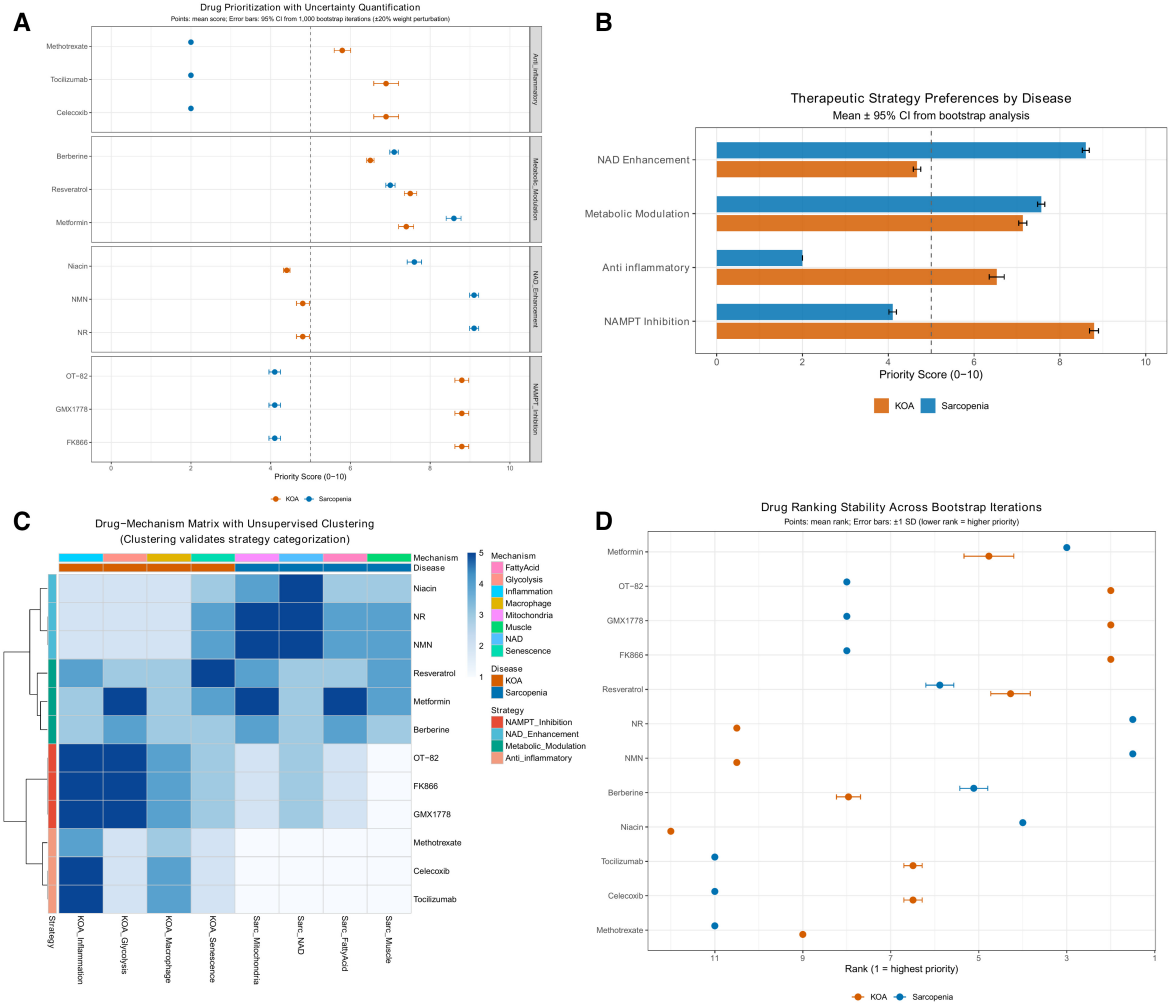
Drug Prioritization with Uncertainty Quantification, Strategy-Level Priority Summaries, Mechanism Clustering, and Bootstrap Ranking Stability for NAMPT-Associated Therapeutic Candidates. Exploratory therapeutic inference analyses based on *NAMPT*-associated molecular signatures; all findings are hypothesis-generating and should not be interpreted as evidence of drug efficacy or clinical recommendation. **(A)** presents a dot plot of candidate drug prioritization scores with uncertainty quantification, stratified by therapeutic strategy category; for each candidate drug, the mean prioritization score is shown with 95% bootstrap confidence intervals, enabling assessment of both magnitude and stability of prioritization. **(B)** shows a disease-level summary of strategy priority scores comparing KOA and sarcopenia, with mean values and 95% bootstrap confidence intervals displayed for each strategy category. **(C)** displays a drug-mechanism matrix with unsupervised hierarchical clustering applied to both drugs and mechanistic features; cell shading reflects association strength, and the clustering is used to assess the coherence of the strategy categorization represented in panels **(A)** and **(B)**. **(D)** presents bootstrap ranking stability summaries across 1,000 resampling iterations; for each candidate drug, the mean rank is shown together with rank variability, where lower rank numbers indicate higher priority. These analyses are exploratory in nature; no causal inference is implied, and findings require prospective experimental and clinical validation before any therapeutic application. *NAMPT*, nicotinamide phosphoribosyltransferase; CI, confidence interval; KOA, knee osteoarthritis; NAD+, nicotinamide adenine dinucleotide

A literature-informed heuristic framework was retained to organize context-specific therapeutic hypotheses. Candidate strategies were categorized as NAD precursor approaches, *NAMPT* modulators, anti-inflammatory agents, metabolic modulators, and sirtuin-related strategies. Scores were generated from the observed association structure, the direction of pathway dysregulation, literature-based mechanistic plausibility, and development context. Candidate-level rankings were summarized with 1,000 bootstrap resamples and 95% percentile confidence intervals, and the exploratory framework is tabulated in Supplementary Table 5 and visualized in (Figure 2).

This framework was used strictly for hypothesis generation. It does not provide evidence of efficacy, treatment recommendations, or proof of target validity, and the bootstrap summaries should not be interpreted as a supported ranking algorithm.

### 2.10 Statistical analysis

All statistical analyses were performed in R. Group comparisons used *t* tests or rank-sum tests according to distributional assumptions. Multiple-testing adjustment was performed with the Benjamini–Hochberg method where appropriate. Nominal *P* values are reported for the three external supporting cohorts to emphasize within-cohort group differences rather than cross-platform pooled inference.

## 3 Results

### 3.1 Cohort characteristics

The discovery stage included four transcriptomic datasets comprising 98 samples in total: 40 synovial samples from KOA-related cohorts and 58 skeletal muscle samples from aging-related proxy cohorts. The external supporting analysis stage added 72 independent samples from three public datasets, including two disease-relevant synovium cohorts and one clinically phenotyped sarcopenia cohort. This design established a clear hierarchy in which abundance discovery, external support, and immune-metabolic contextualization were analytically separated. Technical comparability was highest within the KOA discovery arm because both synovial cohorts used the same Affymetrix platform. By contrast, the skeletal muscle analysis intentionally preserved heterogeneity in phenotype definition so that aging-related proxy cohorts would not be mistaken for clinical sarcopenia. Table 1 summarizes cohort roles, tissues, platforms, and group definitions used in the Frontiers submission package. The clinically phenotyped sarcopenia cohort GSE111016 was defined using Asian Working Group for Sarcopenia criteria, rather than age alone, which distinguishes it from the aging-related skeletal muscle proxy cohorts used in the discovery stage.

**Table 1:** Discovery and external supporting analysis cohorts included in the Frontiers submission.

Cohort	Tissue	Role	Platform	Phenotype definition	Case *n*	Control *n*
GSE55235	Synovium	Discovery	GPL96 microarray	KOA synovium vs. control synovium	10	10
GSE55457	Synovium	Discovery	GPL96 microarray	KOA synovium vs. control synovium	10	10
GSE38718	Skeletal muscle	Discovery	Affymetrix microarray	Aging-related proxy cohort: older vs. younger adults	8	14
GSE25941	Skeletal muscle	Discovery	Affymetrix microarray	Aging-related proxy cohort: older vs. younger women	21	15
GSE12021	Synovium	External support	GPL96 microarray	KOA synovium vs. normal synovium	9	9
GSE254682	Synovium	External support	RNA-seq	KOA synovium vs control synovium	8	7
GSE111016	Vastus lateralis muscle	External support	GPL16791 RNA-seq	AWGS-defined sarcopenia: ALMi (appendicular lean mass index) ≤ 7.00 kg/m^2^ plus low gait speed or low hand grip; vs age-matched older control	19	20

KOA, knee osteoarthritis; RNA-seq, RNA sequencing; GPL96 and GPL16791, GEO platform identifiers; n, number of samples.

### 3.2 Discovery-stage *NAMPT* abundance signal

**Figure 3:**
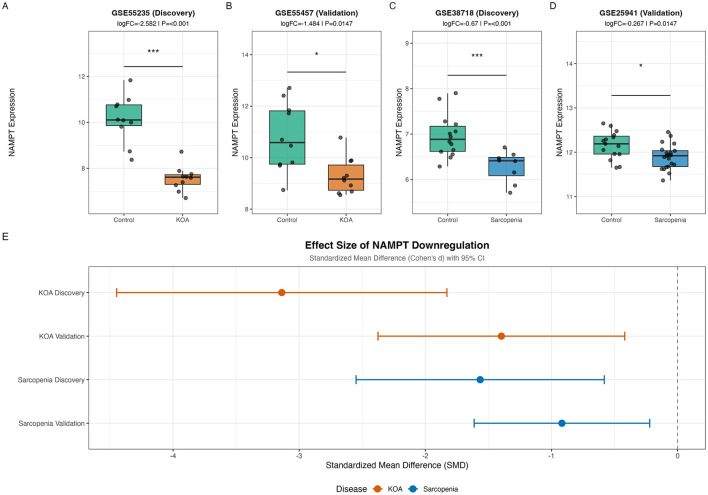
Discovery-stage *NAMPT* abundance signal across KOA synovium and aging-related skeletal muscle proxy cohorts. Multi- summary of the discovery-stage *NAMPT* abundance signal across the four discovery cohorts together with an effect-size overview. **(A)** shows *NAMPT* expression in the KOA synovium cohort GSE55235. **(B)** shows *NAMPT* expression in the KOA synovium cohort GSE55457. **(C)** shows *NAMPT* expression in the aging-related skeletal muscle proxy cohort GSE38718. **(D)** shows *NAMPT* expression in the aging-related skeletal muscle proxy cohort GSE25941. **(E)** summarizes the direction and relative magnitude of *NAMPT* downregulation across the four cohorts as standardized mean differences with 95% confidence intervals, without implying raw-unit equivalence across platforms or tissues. NAMPT expression values are displayed in cohort-specific normalized expression units and should be interpreted within each cohort rather than compared directly across platforms.

Across the four discovery cohorts, *NAMPT* showed a downward abundance signal in both tissue contexts (Figures 3A–E; Supplementary Table 1). In KOA synovium, *NAMPT* was lower in OA than in control samples in both GSE55235 (logFC = −2.58, *P* < 0.001) and GSE55457 (logFC = −1.48, *P* = 0.015). In the aging-related skeletal muscle proxy cohorts, *NAMPT* was also lower in older than in younger samples in GSE38718 (logFC = −0.67, *P* < 0.001) and GSE25941 (logFC = −0.27, *P* = 0.015).

**Figure 4:**
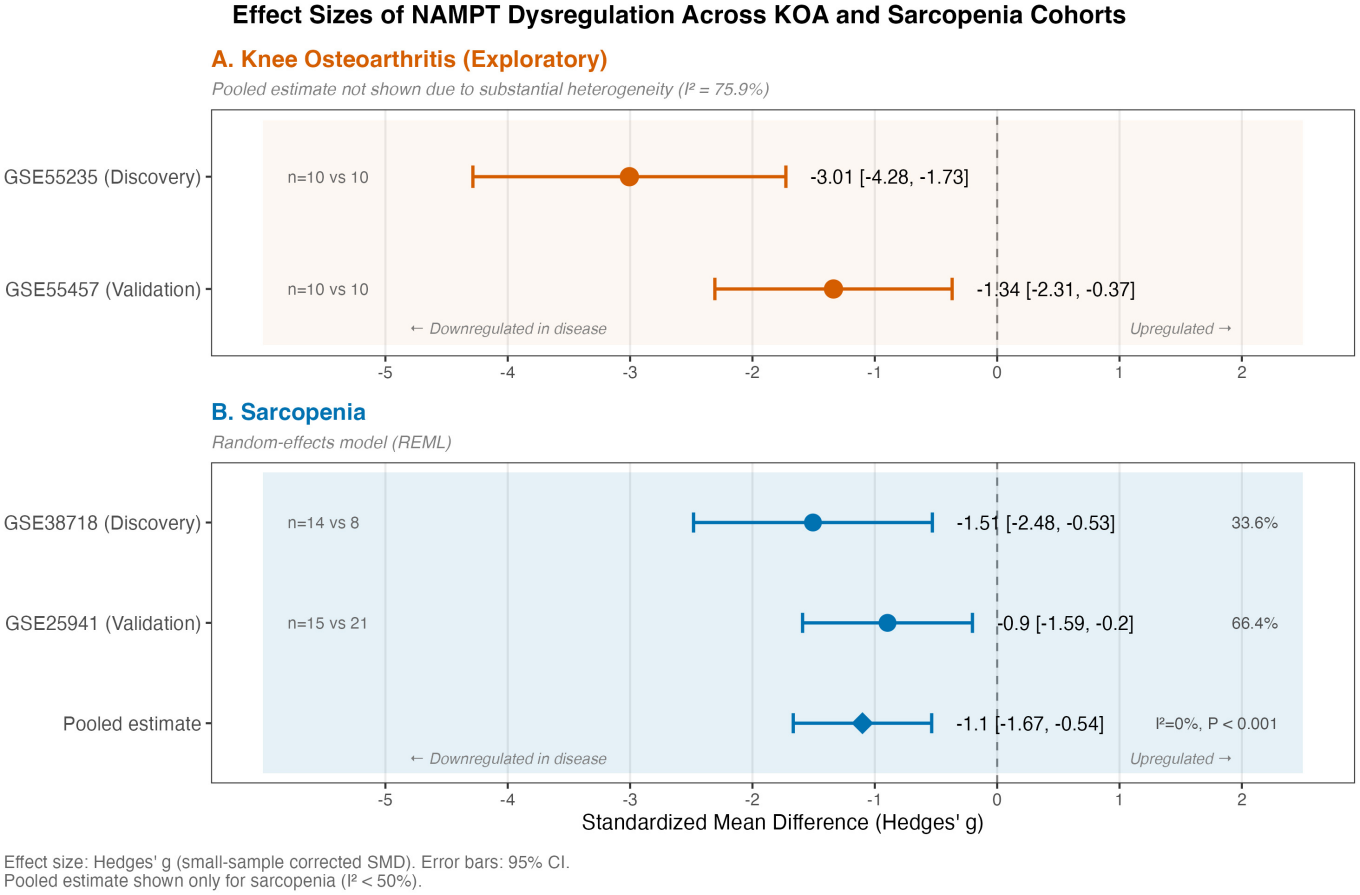
Multi-cohort effect-size synthesis of reduced NAMPT abundance across KOA synovium and aging-related skeletal muscle proxy cohorts. Forest plots illustrating standardized mean differences (SMDs) in *NAMPT* transcript abundance between disease and control groups across independent GEO cohorts, stratified by tissue context. **(A)** displays the meta-analytic forest plot for *NAMPT* expression in knee osteoarthritis (KOA) synovium, incorporating cohorts GSE55235 and GSE55457. Due to substantial between-study heterogeneity (I2 = 75.9%), a pooled summary estimate is not presented for this tissue context; individual cohort effect sizes with 95% confidence intervals are shown separately to preserve interpretive transparency. Each row represents one cohort, and negative SMD values indicate lower *NAMPT* abundance in KOA than in control synovium. **(B)** displays the meta-analytic forest plot for aging-related skeletal muscle proxy cohorts GSE38718 and GSE25941. Individual cohort estimates with 95% confidence intervals are shown together with a random-effects pooled estimate, displayed as a diamond, indicating directionally consistent reduction of *NAMPT* in aging-related muscle relative to younger control muscle. Heterogeneity statistics (I2 and Cochran's Q *p*-value) are reported within each panel. SMD, standardized mean difference; CI, confidence interval; KOA, knee osteoarthritis; GEO, Gene Expression Omnibus. The forest plots are based on discovery-stage cohorts analyzed within cohort; RNA-seq external-support cohorts were analyzed separately and were not included in pooled cross-platform effect-size estimation.

Effect-size synthesis indicated that the muscle proxy signal was directionally stable (Figure 4B; pooled SMD = −1.10, 95% CI −1.67 to −0.54; *P* < 0.001; I^2^ = 0%), whereas the KOA arm showed substantial heterogeneity (Figure 4A; I^2^ = 75.9%) despite both cohorts pointing toward reduced *NAMPT*. Because Figure 4A is presented as independent cohort effect sizes without a pooled diamond, the KOA discovery arm was interpreted as directionally concordant but not quantitatively pooled. Because the KOA discovery arm contained only two datasets, leave-one-cohort-out sensitivity analysis was interpreted qualitatively: after removal of either cohort, the remaining cohort still showed lower *NAMPT* in OA synovium, indicating directional stability despite uncertainty in pooled magnitude. These findings support a discovery-stage abundance signal while also arguing against overstatement of universal stability, particularly in the muscle-side analysis, where aging-related proxy cohorts and clinically phenotyped sarcopenia cohorts capture related but non-identical biological states.

The wide confidence interval in the KOA discovery arm likely reflects between-cohort heterogeneity and modest sample size. Possible sources include disease-stage variation, synovial sampling differences, control-source differences, and inflammatory burden. Although K–L grade may be relevant, sample-level K–L metadata were not consistently available across the included public datasets, preventing reliable K–L-stratified analysis.

### 3.3 External cohort support for *NAMPT* abundance differences

**Figure 5:**
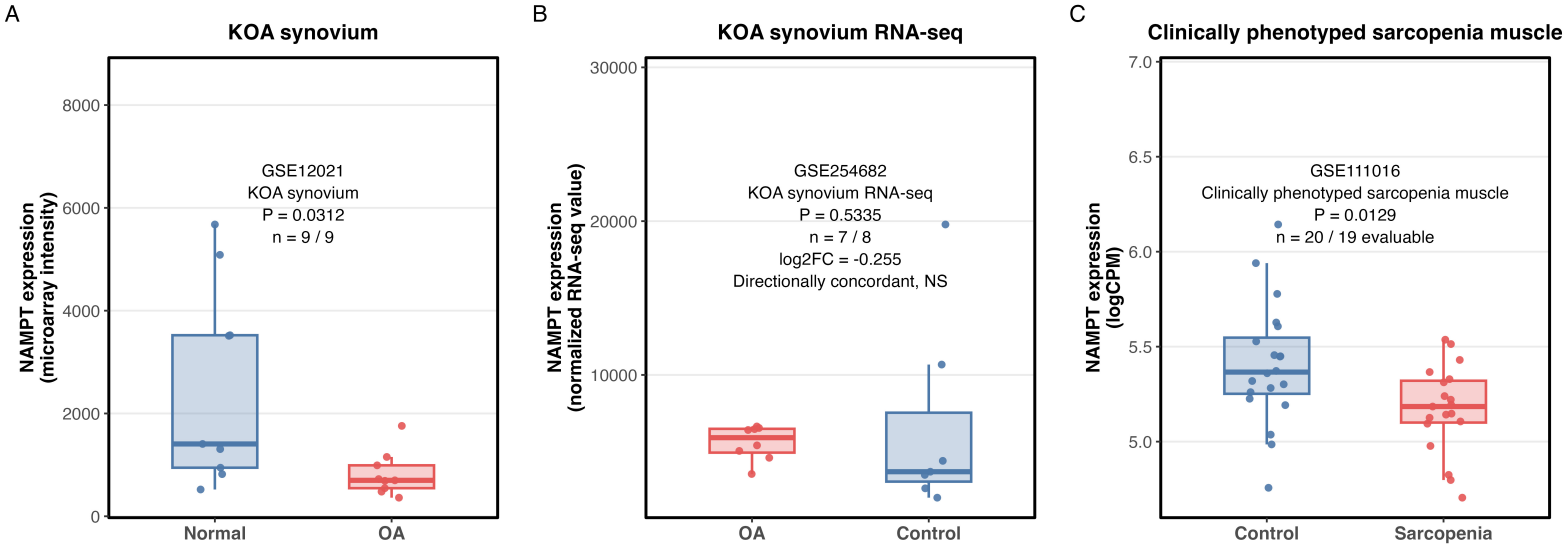
External cohort support for NAMPT abundance differences in independent public cohorts Box-and-dot plots show *NAMPT* expression in three independent public cohorts used for external supporting analysis. **(A)** shows the disease-relevant synovium microarray cohort GSE12021, in which *NAMPT* was lower in knee osteoarthritis (OA) synovium than in normal synovium. **(B)** shows the disease-relevant synovium RNA-seq cohort GSE254682, in which *NAMPT* remained directionally concordant with the primary analysis but was not statistically significant. **(C)** shows the clinically phenotyped sarcopenia cohort GSE111016, in which *NAMPT* was lower in sarcopenic vastus lateralis muscle than in control muscle. Exact *P* values and sample counts are shown within each panel. Platform types were GPL96 microarray for GSE12021, RNA-seq for GSE254682, and GPL16791 RNA-seq for GSE111016.

We next assessed *NAMPT* expression in independent public cohorts selected to provide disease-relevant external support rather than classifier-style performance analysis (Figure 5; Supplementary Table 6). In the independent KOA synovium microarray cohort GSE12021, *NAMPT* expression was significantly lower in osteoarthritic synovium than in normal synovium (mean 822.84 vs 2,532.97; *P* = 0.0312). In the cross-platform KOA synovium RNA-seq cohort GSE254682, *NAMPT* again showed a downward direction in OA (mean 5,593.77 vs 6,674.44; log2FC = −0.255), although this difference was not statistically significant (*P* = 0.5335). In the muscle-side external support analysis, GSE111016 represented a clinically phenotyped sarcopenia cohort and showed lower NAMPT expression in sarcopenic vastus lateralis muscle than in control muscle (mean 5.171 vs. 5.403 logCPM; *P* = 0.0129).

Taken together, these external cohorts provide partial support for reduced NAMPT abundance in disease-relevant musculoskeletal contexts. The support was strongest in GSE12021 and GSE111016, whereas GSE254682 was directionally concordant but statistically inconclusive. Therefore, these cohorts should be interpreted as external support for selected abundance patterns rather than as full validation of the discovery-stage immune-metabolic architecture.

The non-significant result in GSE254682 is important for interpretation. It suggests that the synovial NAMPT abundance signal may not be uniformly reproducible across all KOA synovial cohorts. Several factors may contribute to this heterogeneity, including differences in synovial sampling, disease-stage distribution, inflammatory burden, control-tissue definition, RNA-seq versus microarray quantification, and the modest sample size of GSE254682. Because sample-level clinical and sampling metadata were not uniformly available across cohorts, these potential sources of heterogeneity could not be formally stratified. We therefore interpret GSE254682 as directionally concordant but statistically inconclusive, rather than as definitive external validation.

### 3.4 Distinct immune and metabolic contexts characterize synovial and skeletal muscle cohorts

**Figure 6:**
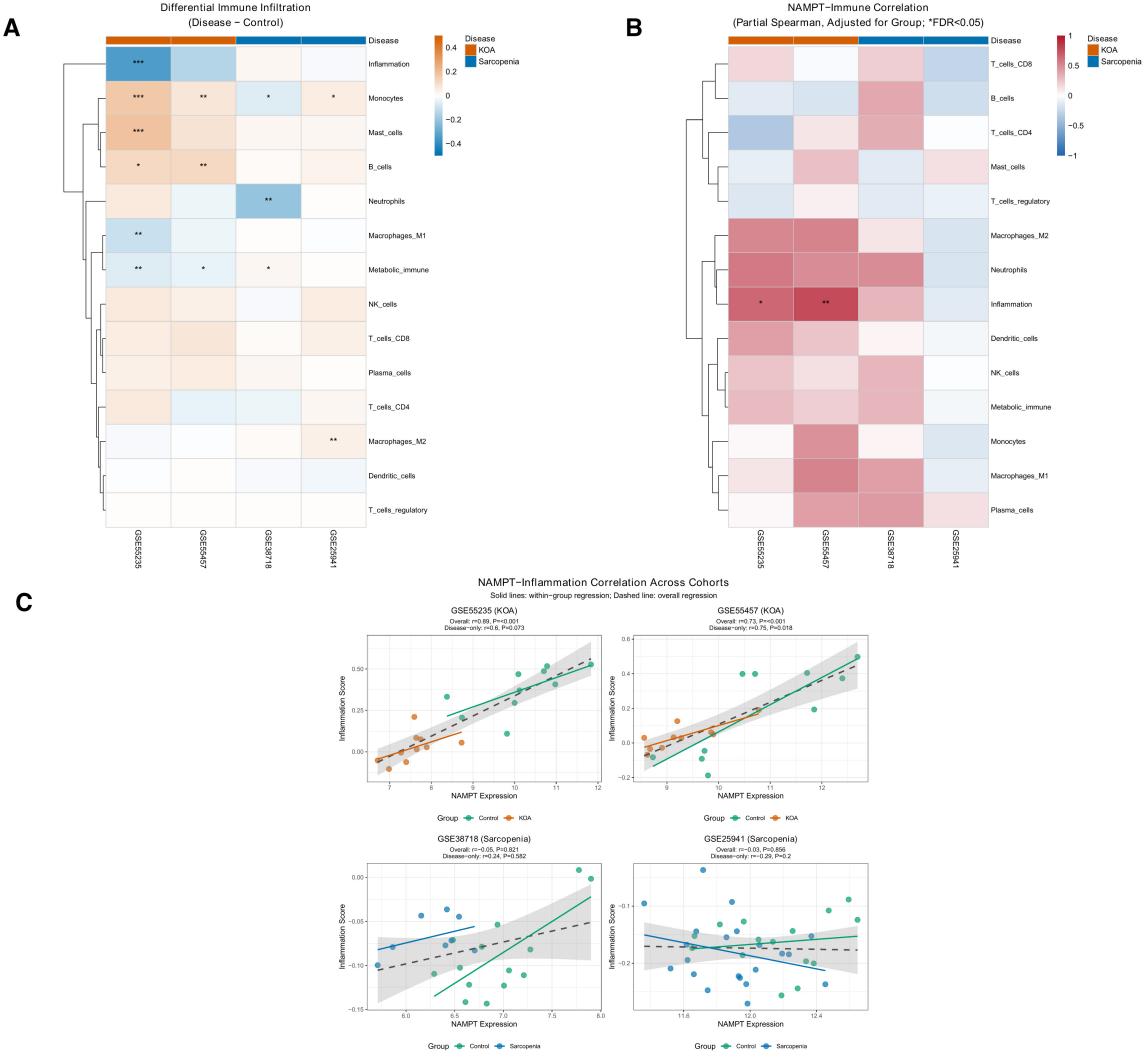
Disease-Minus-Control Immune Landscape and Disease-Adjusted NAMPT-Immune Partial Correlations Across Discovery Cohorts, with Cohort-Level Representative Inflammation Scatter Plots. Characterization of immune-feature differences and *NAMPT*-linked immune association structure across the four discovery cohorts. **(A)** presents a heatmap of disease-minus-control differences in ssGSEA-derived immune cell enrichment scores across GSE55235, GSE55457, GSE38718, and GSE25941, with rows representing immune cell populations and columns representing cohorts; positive values (warmer colors) indicate enrichment in disease relative to control. **(B)** displays a heatmap of disease-adjusted partial correlation coefficients between *NAMPT* expression and immune-feature scores across the same four discovery cohorts, with case-control status included as a covariate; color intensity reflects the magnitude and direction of the partial correlation, and asterisks denote features meeting the false discovery rate threshold shown in the figure. **(C)** shows a representative scatter plot for GSE55235 depicting the relationship between *NAMPT* expression and the inflammation score. **(D)** shows the corresponding GSE55457 scatter plot. **(E)** shows the corresponding GSE38718 scatter plot. **(F)** shows the corresponding GSE25941 scatter plot. In panels **(C–F)**, each point represents one sample, within-group regression lines are overlaid, the dashed line shows the overall trend, and cohort-specific correlation statistics are annotated. Taken together, **(A–F)** show that discovery-stage immune-feature landscapes and *NAMPT*-inflammation relationships differ in strength and consistency between KOA synovium and aging-related skeletal muscle proxy cohorts. ssGSEA, single-sample Gene Set Enrichment Analysis; *NAMPT*, nicotinamide phosphoribosyltransferase; KOA, knee osteoarthritis; FDR, false discovery rate. In panel **(A)**, the color scale represents disease-minus-control differences in ssGSEA-derived immune enrichment scores; in panel **(B)**, the color scale represents disease-adjusted partial Spearman correlation coefficients (ρ).

**Figure 7:**
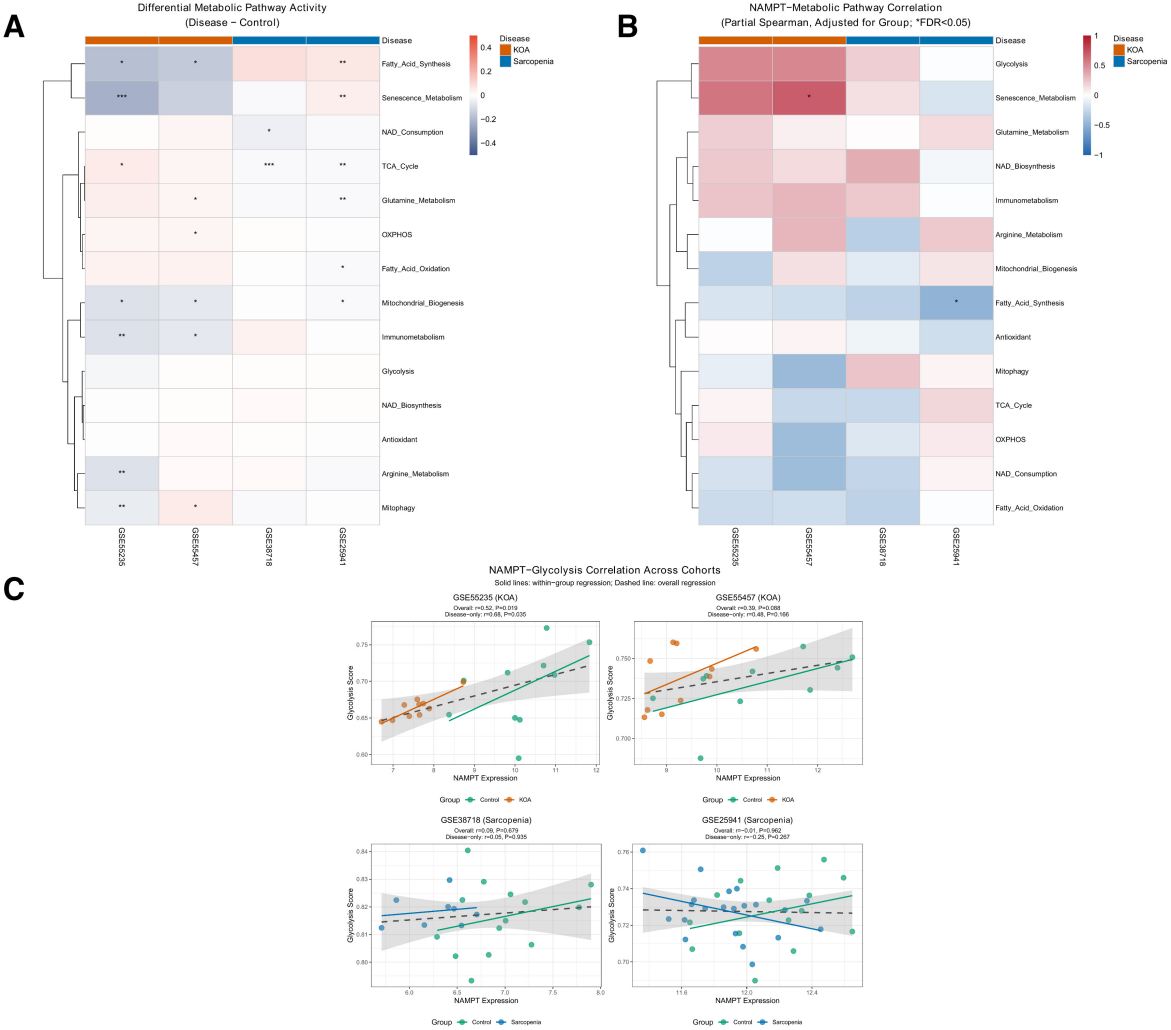
Disease-Minus-Control Metabolic Landscape and Disease-Adjusted NAMPT-Metabolic Partial Correlations Across Discovery Cohorts, with Cohort-Level Representative Glycolysis Scatter Plots. Characterization of the metabolic pathway landscape across the four discovery cohorts and its relationship to *NAMPT* expression, presented in structural parallel with Figure 6 to facilitate cross-tissue comparison. **(A)** presents a heatmap of disease-minus-control differences in ssGSEA-derived metabolic pathway enrichment scores across GSE55235, GSE55457, GSE38718, and GSE25941, with rows representing metabolic gene sets and columns representing cohorts; color indicates direction and magnitude of enrichment relative to control tissue. **(B)** displays a heatmap of disease-adjusted partial correlation coefficients between *NAMPT* expression and metabolic pathway scores across the same four discovery cohorts, with case-control status included as a covariate; asterisks indicate features meeting the false discovery rate threshold shown in the . **(C)** shows a representative scatter plot for GSE55235 depicting the relationship between *NAMPT* expression and the glycolysis score. **(D)** shows the corresponding GSE55457 scatter plot. **(E)** shows the corresponding GSE38718 scatter plot. **(F)** shows the corresponding GSE25941 scatter plot. In s **(C–F)**, each point represents one sample, within-group regression lines are overlaid, the dashed line shows the overall trend, and cohort-specific correlation statistics are annotated. Comparison of Figures 5 and 6 shows that *NAMPT*-associated inflammatory relationships are stronger in KOA synovium, whereas metabolic pathway relationships are more attenuated and context-dependent in the aging-related skeletal muscle proxy cohorts. add ssGSEA, single-sample Gene Set Enrichment Analysis; NAMPT, nicotinamide phosphoribosyltransferase; FDR, false discovery rate. In panel **(A)**, the color scale represents disease-minus-control differences in ssGSEA-derived metabolic pathway enrichment scores; in panel **(B)**, the color scale represents disease-adjusted partial Spearman correlation coefficients (ρ).

Immune and metabolic feature scoring across the four discovery cohorts (Figures 6A and B, Figures 7A and B, and Supplementary Tables 2 and 3) showed that the two tissue contexts differed more in their surrounding network state than in the direction of NAMPT abundance alone. KOA synovium displayed a more inflammatory landscape, enriched for macrophage- and dendritic-cell-related signatures and for higher inflammatory pathway scores, whereas the aging-related skeletal muscle proxy cohorts showed weaker immune signals and a broader pattern suggestive of metabolic impairment, including altered oxidative and mitochondrial programs rather than overtly inflamed transcriptomic organization.

These findings support the revised interpretation that synovium and muscle should be viewed as distinct immune-metabolic contexts. Even when *NAMPT* changes in the same direction, the biological programs surrounding that change are not equivalent across the two tissues. Because these analyses were performed using bulk tissue transcriptomes, the observed immune and metabolic feature differences should be interpreted as tissue-level transcriptomic contexts rather than cell-type-resolved mechanisms. The present data cannot determine whether reduced NAMPT expression reflects cell-intrinsic changes in synovial fibroblasts or muscle fibers, altered proportions of macrophages or other stromal/immune populations, or a combination of both.

### 3.5 Disease-adjusted *NAMPT* coupling

Disease-adjusted association analyses, summarized across all four discovery cohorts in Figure 6B and Figure 7B and illustrated with representative cohort-level scatter plots in Figures 6C–F and Figures 7C–F, sharpened this contrast. Full cohort-level outputs are reported in Supplementary Table 4. In KOA synovium, NAMPT retained positive partial correlations with inflammatory signatures (partial r range 0.64–0.77, FDR < 0.05) and with glycolytic activity (partial *r* = 0.52, FDR < 0.05), indicating that the residual NAMPT signal remained embedded in an inflammatory-glycolytic association structure even after accounting for group status. In the aging-related skeletal muscle proxy cohorts, these inflammatory associations were absent or markedly attenuated, and the remaining associations were more consistent with metabolic stress than with an active immune-linked program.

Fisher's z testing confirmed that the between-context difference in association structure was statistically meaningful, particularly for *NAMPT*-inflammation relationships. This supports a tissue-aware, discovery-stage systems interpretation rather than treating a similar direction of abundance change as biologically equivalent across tissues.

Cohort-stratified discovery-stage analyses further indicated that the coupling signal was not evenly distributed across datasets but showed a tissue-weighted pattern. *NAMPT*-inflammation coupling remained positive in both KOA cohorts (GSE55235 partial *r* = 0.64, 95% CI 0.28 to 0.84; GSE55457 partial *r* = 0.77, 95% CI 0.48 to 0.91), whereas the aging-related skeletal muscle proxy cohorts showed attenuated and heterogeneous inflammation coupling (GSE38718 partial *r* = 0.30, 95% CI −0.15 to 0.64; GSE25941 partial *r* = −0.13, 95% CI– 0.44 to 0.21). A similar pattern was observed for glycolysis and senescence-associated metabolic coupling, which remained directionally positive in both KOA cohorts but were weak or absent in the muscle proxy cohorts (Supplementary Table 7; Supplementary Figure 4). These cohort-level differences explain why the discovery heatmaps in Figures 6 and 7 display all four cohorts while still supporting a tissue-weighted rather than tissue-agnostic interpretation.

A reduced external association analysis showed observed patterns with partial directional consistency in selected follow-up cohorts. In the KOA synovium RNA-seq cohort GSE254682, NAMPT retained a positive disease-adjusted association with the inflammatory response module (partial rho = 0.73, 95% CI 0.32 to 0.91; *P* = 0.0031), whereas glycolysis remained directionally positive but non-significant (partial rho = 0.41; *P* = 0.1401). In the clinically phenotyped sarcopenia cohort GSE111016, inflammatory and glycolytic associations were attenuated or negative (partial rho = −0.22 and −0.19, respectively), whereas oxidative phosphorylation showed a positive association with NAMPT (partial rho = 0.46, 95% CI 0.16 to 0.68; *P* = 0.0040). These reduced external analyses do not reproduce the full discovery-stage architecture. They should therefore be interpreted as partial module-level consistency with the discovery-stage pattern, rather than as validation of distinct tissue-specific association structures. (Supplementary Table 8; Supplementary Figure 5).

### 3.6 Exploratory therapeutic implications

Exploratory therapeutic inference was retained only as a hypothesis-oriented extension of the coupling analysis. Figure 2 summarizes candidate prioritization, disease-level strategy priority scores, mechanism clustering, and bootstrap ranking stability, while Supplementary Table 5 provides the underlying exploratory framework. Within the predefined heuristic framework, *NAMPT* inhibition scored higher in the KOA synovial context, whereas NAD-restoration strategies scored higher in the skeletal muscle proxy context. These rankings should not be interpreted as evidence of efficacy. Instead, they organize different experimental questions that may follow from inflamed synovium vs. metabolically impaired muscle.

Secondary cohort-level separation analyses were moved out of the main validation narrative and retained only as supplementary descriptive results.

## 4 Discussion

### 4.1 Principal findings

**Figure 8:**
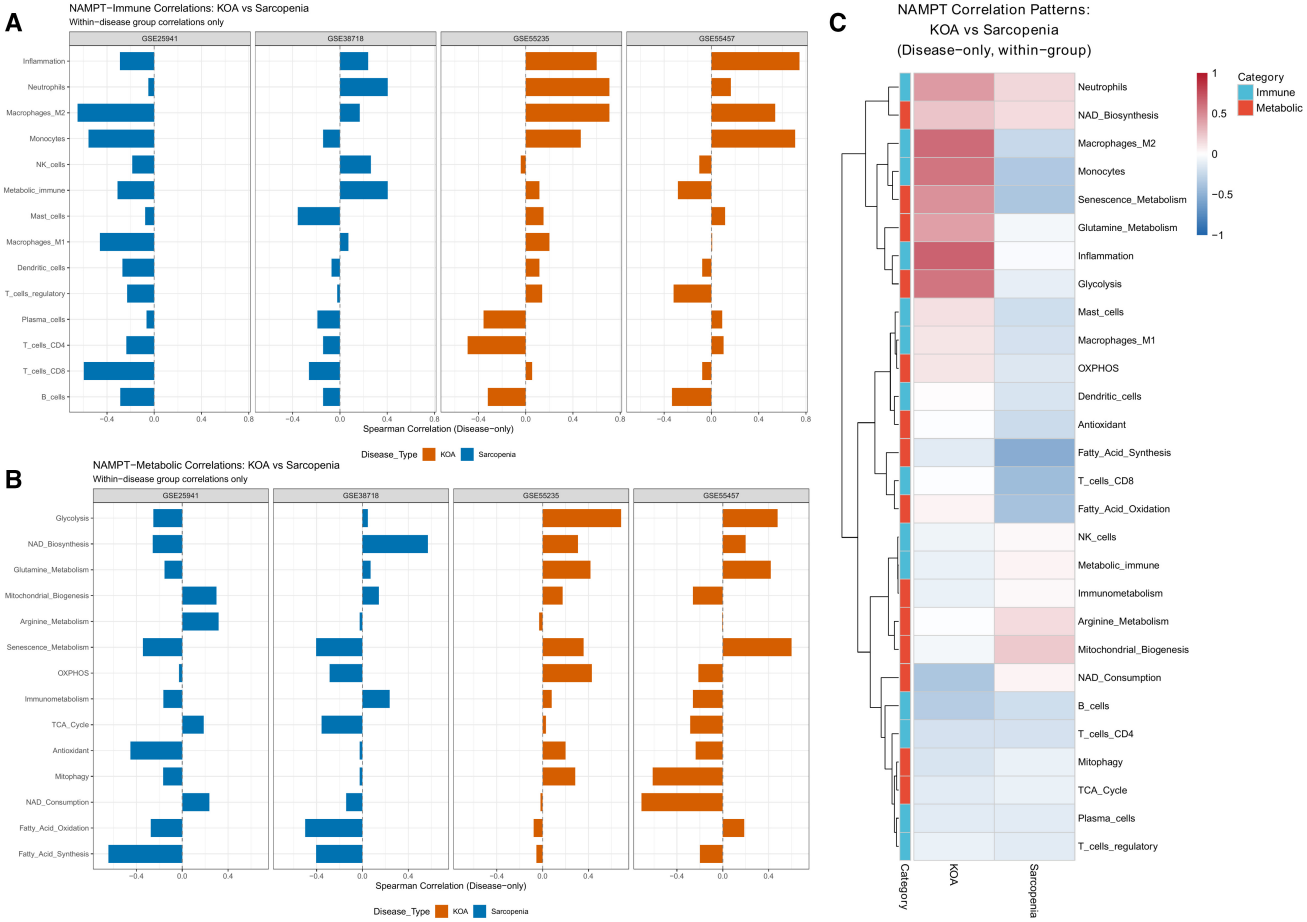
Within-Disease Cohort-Faceted Immune and Metabolic Correlation Summaries and Cross-Condition Clustered Comparison of NAMPT Associations. Summary of disease-only within-group correlation analyses and integrative cross-condition comparison. **(A)** presents cohort-faceted summaries of within-disease Spearman correlation coefficients between *NAMPT* expression and immune-feature scores computed exclusively within disease samples for GSE25941, GSE38718, GSE55235, and GSE55457. Feature identity is shown on the y-axis, correlation magnitude is shown on the x-axis, and cohorts are displayed in separate facets to preserve cohort-level structure. **(B)** presents the corresponding within-disease metabolic correlation summary, structured identically to **(A)**, enabling direct assessment of metabolic correlates of *NAMPT* independently of disease-control contrast. **(C)** displays a clustered heatmap in which rows represent selected immune and metabolic features and columns represent condition-level averages for KOA versus muscle proxy condition-level NAMPT correlation patterns. Unsupervised hierarchical clustering of both rows and columns is used to reveal whether KOA and sarcopenia share or diverge in their *NAMPT*-associated molecular signatures. This figure summarizes the same within-disease correlation structure visualized in **(A)** and **(B)** and does not introduce new statistical tests beyond those reported in Figures 6 and 7. *NAMPT*, nicotinamide phosphoribosyltransferase; KOA, knee osteoarthritis. In panel **(C)**, the heatmap color scale represents Spearman correlation coefficients (ρ).

This revised analysis supports two related but distinct conclusions, with different levels of evidential strength. First, reduced NAMPT abundance received partial external support, most clearly in GSE12021 and GSE111016, while the RNA-seq KOA synovium cohort GSE254682 was directionally concordant but statistically inconclusive. Second, the tissue-dependent immune-metabolic association structure should be interpreted primarily as a discovery-stage pattern, with only limited external corroboration in reduced module-level analyses. The main contribution of the study is therefore not simply that *NAMPT* declines in more than one musculoskeletal context, but that a similar direction of abundance change can coexist with distinct tissue-dependent association patterns, with greater apparent stability in KOA than in aging-related skeletal muscle proxy cohorts. A reduced external association analysis showed limited module-level consistency with this contrast, including preserved inflammatory linkage in external KOA synovium and a more oxidative-phosphorylation-oriented pattern in clinically phenotyped sarcopenia muscle. Cohort-faceted within-disease summaries and the condition-level clustered comparison in Figure 8 were consistent with this tissue-dependent interpretation rather than a single shared *NAMPT*-associated profile.

### 4.2 Synovial heterogeneity and the non-significant RNA-seq cohort

The non-significant NAMPT difference in GSE254682 warrants particular caution. Although the direction of change was consistent with the microarray synovial cohorts, the absence of statistical significance indicates that the synovial NAMPT signal is sensitive to cohort context. Public KOA synovial datasets may differ in sampling site, disease stage, inflammatory activity, surgical context, control-tissue source, and sequencing or normalization procedures. These factors are biologically relevant because recent cellular characterization and single-cell studies emphasize that OA synovium is a heterogeneous tissue containing variable proportions of fibroblasts, macrophages, endothelial cells, and other immune or stromal populations (51–53). These findings reinforce the need for cell-resolved follow-up of bulk transcriptomic signals. Therefore, the GSE254682 result reduces confidence in a uniformly reproducible synovial abundance effect and supports a more cautious interpretation focused on partial external support rather than definitive replication.

### 4.3 Why tissue context matters immunologically

The synovium is an inflammatory stromal niche in which activated fibroblasts, macrophages, and other infiltrating immune populations participate directly in pathology (54–59). In this setting, reduced *NAMPT* abundance does not imply biological irrelevance. The residual *NAMPT* signal still tracks with inflammatory and glycolytic programs. This pattern is compatible with the view that the synovial environment remains immunometabolically active even when individual metabolic enzymes show reduced transcript abundance.

Skeletal muscle, by contrast, is dominated by energetic maintenance, contractile function, and mitochondrial quality control (60–66). Recent reviews of skeletal muscle NAD+ salvage and NAD restoration in aging further reinforce the relevance of mitochondrial resilience, metabolic flexibility, and muscle maintenance in this tissue context (30, 31). When *NAMPT* declines in this context, the surrounding association structure points less toward coordinated inflammatory activation and more toward metabolic vulnerability. Treating these two contexts as equivalent would therefore obscure a meaningful systems-level difference.

### 4.4 Bulk-tissue interpretation and unresolved cell-type origin

The present study cannot assign NAMPT changes to specific cell types. In OA synovium, NAMPT variation could reflect altered expression within synovial fibroblasts or macrophages, changes in the relative abundance of lining-layer or immune-cell populations, or both. Similarly, in skeletal muscle, lower NAMPT abundance could reflect myofiber-intrinsic metabolic changes, altered proportions of non-myofiber cell populations, or differences in tissue composition. Although public single-cell references and deconvolution approaches may help address this issue in future work, such analyses require careful matching of tissue source, disease stage, platform, and cell-state definitions. Therefore, the present findings should be interpreted as bulk tissue-level association patterns rather than cell-type-specific mechanisms. Until matched single-cell resolution data become available, the cellular origin of the observed NAMPT transcript changes—whether reflecting altered expression within resident cells, shifts in cell-type composition, or both—remains to be elucidated.

### 4.5 Relationship between aging-related muscle proxy findings and clinical sarcopenia

An important interpretive issue is that the discovery-stage muscle cohorts and the external sarcopenia cohort do not represent identical phenotypes. GSE38718 and GSE25941 were analyzed as aging-related skeletal muscle proxy cohorts, whereas GSE111016 was interpreted as a clinically phenotyped sarcopenia cohort. Chronological muscle aging and clinical sarcopenia are biologically related but not interchangeable. Aging-related transcriptomic datasets may capture broad changes in mitochondrial function, metabolic flexibility, exercise responsiveness, and tissue maintenance, whereas clinical sarcopenia reflects a phenotypic state involving impaired muscle mass, strength, and/or physical performance.

Therefore, the metabolic-deficit-oriented NAMPT association pattern observed in the aging-related proxy cohorts should not be interpreted as direct evidence of the same mechanism in clinical sarcopenia. Rather, the lower NAMPT expression observed in GSE111016 suggests that NAMPT reduction may be shared across aging-related muscle decline and clinical sarcopenia, but it does not establish that the discovery-stage association structure is operative in sarcopenia pathology. We therefore interpret the muscle-side findings as indicating possible overlapping metabolic vulnerability, not as proof of a single unified sarcopenia mechanism. This distinction is clinically relevant because GSE111016 was not an age-only comparison cohort; sarcopenia was defined using appendicular lean mass index together with gait speed or grip strength according to Asian Working Group for Sarcopenia criteria. Importantly, the lower NAMPT expression observed in GSE111016 should not be interpreted as pathophysiological validation of the discovery-stage aging-related proxy findings. Instead, the aging-related proxy cohorts and clinically phenotyped sarcopenia cohort likely represent related but distinct biological states. Their shared directional abundance signal may therefore indicate partial convergence rather than direct biological equivalence.

### 4.6 Protein-layer contextualization

The protein layer is best viewed here as contextualization rather than direct matched validation. Recent eNAMPT-focused literature further supports this distinction by emphasizing that extracellular NAMPT has biological functions that are not equivalent to intracellular transcript abundance (27). OA studies often report elevated extracellular *NAMPT*/visfatin in serum, synovial fluid, or inflammatory tissue compartments, whereas muscle studies link impaired NAD salvage capacity to reduced intracellular *NAMPT*-related support and show exercise responsiveness of the salvage pathway (24, 25, 28, 29, 54, 56, 62, 66). These observations are not inconsistent with the present transcriptomic results. Rather, they support a compartment-specific interpretation: local transcript depletion in tissue-resident cells may coexist with elevated extracellular inflammatory *NAMPT*, especially in chronically inflamed synovium.

This distinction strengthens the argument that i*NAMPT*-centered and e*NAMPT*-centered biology should not be collapsed into a single abundance narrative when interpreting musculoskeletal disease.

### 4.7 Implications for immune-metabolic interpretation

The systems-level implication is that uniform extrapolation should be avoided. A broad NAD-boosting strategy may interact differently with a synovial transcriptomic state that remains linked to inflammation than with a muscle state that appears more metabolically depleted than immune-linked, especially in light of recent discussions of NAD+ salvage and NAD restoration in skeletal muscle aging (30, 31, 60, 64, 67). The exploratory strategy-level summaries and ranking-stability displays in Figure 2 are therefore best interpreted as organizational, hypothesis-generating outputs rather than treatment guidance. The present study does not justify treatment recommendations, but it does support context-specific experimental design rather than tissue-agnostic reasoning. Although recent drug-discovery literature highlights continued interest in NAMPT modulators, the present therapeutic ranking framework remains exploratory and should not be interpreted as evidence of efficacy or treatment recommendation (68).

### 4.8 Limitations

Several limitations are important for a Frontiers-oriented interpretation. A major limitation is that the reduced external module analysis did not recapitulate the full discovery-stage association architecture. Because only simplified hallmark-based modules could be applied consistently in the external cohorts, these results should be interpreted as partial directional consistency rather than external validation of the co-expression or immune-metabolic network structure. First, this is a bulk transcriptome analysis and therefore cannot resolve whether NAMPT changes arise from specific synovial fibroblast, macrophage, myofiber, or stromal populations, nor can it capture the broader cell-resolved immune and stromal architecture highlighted by recent cellular and single-cell studies of knee OA (51–53). Second, the external supporting analysis layer mainly supports abundance direction and group difference; it does not reproduce the full coupling architecture because pathway-level harmonization was not available across all external-support datasets. Third, the reduced external association analysis used simplified hallmark-based modules rather than the exact discovery-stage feature framework. Fourth, heterogeneity on the muscle side remains substantial because aging-related proxy cohorts and clinical sarcopenia cohorts represent overlapping but distinct states. As a result, the discovery-stage metabolic association pattern derived from aging-related proxy cohorts cannot be assumed to represent the full pathology of clinical sarcopenia. Fifth, the protein-layer support is literature-based and compartment-specific rather than derived from matched transcript-protein measurements in the same samples. In particular, the RNA-seq synovial cohort GSE254682 showed the same downward direction for NAMPT but did not reach statistical significance. This non-significant result constrains the strength of the synovial external support and indicates that reduced NAMPT abundance cannot be claimed as uniformly replicated across all KOA synovial datasets.

Additional limitations include modest cohort sizes, residual heterogeneity across public datasets, and the exploratory nature of the therapeutic ranking framework. For the KOA synovial datasets, sample-level Kellgren–Lawrence grade and detailed synovial sampling metadata were not consistently available, preventing formal stratified analyses of whether NAMPT effect size varied by radiographic severity or sampling location. Public-data confounding, including incomplete control of age, body composition, medication exposure, and cohort-source effects, cannot be fully excluded. Reverse causality also remains unresolved, and the condition-level separation visualized in Figure 8 should not be interpreted as proof that cohort-origin effects are absent. Finally, transcript abundance should not be equated with protein-level or compartment-specific e*NAMPT* and i*NAMPT* activity. These features justify treating the present study as a systems-level, hypothesis-generating analysis with external support for the abundance signal rather than as definitive translational proof.

## 5 Conclusions

Reduced NAMPT abundance showed partial external support in selected disease-relevant cohorts, with statistically supported findings in GSE12021 and GSE111016 and directionally concordant but statistically inconclusive evidence in GSE254682. In the muscle-side analysis, the aging-related discovery cohorts and the clinically phenotyped sarcopenia cohort should be interpreted as related but non-equivalent contexts. The tissue-dependent immune-metabolic association patterns observed in the discovery stage should be interpreted as hypothesis-generating observed patterns with partial module-level consistency in follow-up cohorts, rather than as externally validated network structures.

## Data Availability

The datasets analyzed in this study are publicly available in the NCBI Gene Expression Omnibus under accession numbers GSE55235, GSE55457, GSE38718, GSE25941, GSE12021, GSE254682, and GSE111016. The original contributions presented in the study are included in the article and Supplementary material. Further inquiries can be directed to the corresponding authors.
